# The Impact of Malnutrition and Multimodal Prehabilitation on Quality of Life in Head and Neck Cancer Patients Following Resection and Microvascular Reconstruction: A Cross-Sectional Study

**DOI:** 10.3390/jcm15083050

**Published:** 2026-04-16

**Authors:** Benjamin Walch, Alexander Gaggl, Katharina Zeman-Kuhnert, Valentina Ranstl, Martin Geroldinger, Birgit Mitter, Anna Lena Bridts, Gian Battista Bottini, Florian Huber

**Affiliations:** 1 Department of Oral and Maxillofacial Surgery and Center for Reconstructive Surgery, Paracelsus Medical University, 5020 Salzburg, Austria; 2 Team Biostatistics and Big Medical Data, IDA Lab Salzburg, Paracelsus Medical University, 5020 Salzburg, Austria; 3 Research Program Biomedical Data Science, Paracelsus Medical University, 5020 Salzburg, Austria

**Keywords:** head and neck squamous cell carcinomas, cancer, prehabilitation, nutrition therapy, speech therapy, psychology

## Abstract

**Background:** Dysphagia and malnutrition are common among head and neck squamous cell carcinoma (HNSCC) patients. Evidence and guidelines emphasize treatment and prevention of these conditions before surgery. In this context, multimodal prehabilitation (MP) is an essential part of a holistic treatment approach. However, the specific components and their relative importance remain debated. This study aimed to evaluate the impact of nutritional, logopedic and psychological MP. **Methods:** Adult HNSCC patients who completed the German version of the Functional Assessment of Cancer Therapy—Head and Neck (FACT-H&N) quality-of-life (QOL) questionnaire after undergoing curative resection and reconstructive surgery were included in this cross-sectional study. Clinical data on psychological, logopedic and nutritional MP and possible confounders was collected. To evaluate the nutritional status, bodyweight loss, the body mass index (BMI) and the Graz Malnutrition Screening (GMS) score were recorded. We determined the length of stay (LOS), the QOL, the Clavien–Dindo type III and IV complication rate (CR) as the outcome parameters for MP. **Results:** In total, 102 patients were included. Of those, 68 were male, while the other 34 were female. The mean age was 59.82 ± 12.27 years. The average GMS was 3.11 ± 1.45. Simultaneously, 62.75% of patients were at risk or malnourished. Malnutrition was significantly associated with adverse outcomes in the univariate, but only with decreased QOL in the multivariate model. On the other hand, MP was significantly associated with reduced LOS and improved QOL. These findings remained robust even after adjustment for possible confounders. Neither had a significant effect on the CR. **Conclusions:** Our findings suggest that malnutrition is a potential risk factor for adverse outcomes in curative HNSCC therapy. The GMS is a sensitive tool for identifying patients at risk of malnutrition in HNSCC surgery prehabilitation. Our multimodal protocol was associated with improved postoperative outcomes following curative surgical resection and free flap reconstruction. The observed associations may reflect potential synergistic interactions within the multimodal framework.

## 1. Introduction

Locally advanced head and neck squamous cell carcinoma (HNSCC) significantly impairs vital functions essential for daily life. These include swallowing, breathing, speaking, phonation, aesthetics and expression. Consequently, these impairments reduce patients’ functional capacity, psychological and physical well-being, and social interactions.

The burden of disease on patients’ well-being can be quantified using quality-of-life (QOL) questionnaires. Common Sense Oncology and the European Organisation for Research and Treatment of Cancer underscore the importance of employing QOL metrics in clinical research to weigh the benefits and harm of a therapy and ensure value assessment of cancer treatments [1].

Even in cases where a curative treatment strategy is pursued, both the disease itself and the associated surgical and adjuvant therapies may result in additional functional impairments, deformities, and disabilities. Free flap reconstructive procedures following tumor resection not only improve overall survival rates but also play a critical role in restoring lost functions and preserving QOL [2,3,4]. These procedures allow surgeons to perform radical cancer resections while minimizing the impact on the patient’s functional and aesthetic outcomes.

However, managing head and neck defects remains one of the most complex challenges in reconstructive surgery [5].

Treatment is further complicated by the fact that a significant proportion of patients can be characterized by advanced age, with multiple comorbidities, malnutrition, cachexia, sarcopenia or a history of tobacco and alcohol addiction. Those clinical conditions have been identified as predictors of adverse outcomes in cancer and reconstructive surgery [6,7].

In particular, cachexia, sarcopenia and malnutrition are highly prevalent among HNSCC patients. These conditions are similar and somewhat overlap. Cancer cachexia is defined as a multifactorial syndrome characterized by a progressive loss of skeletal muscle tissue, with or without a loss of body fat that is at least partially irreversible with nutritional therapy and can lead to increasing functional disability [8,9]. On the other hand, sarcopenia is the pathological loss of muscle mass and strength with risk of adverse outcomes and malnutrition induced by too much, too little or imbalanced intake of nutrients [8,10,11]. Cancers may cause any or all of these conditions. They represent a common endpoint of underlying pathological processes and thus are closely related. Systemic inflammation caused by cancer leads to muscle proteolysis and the presence of swallowing disorders and odynophagia impairs oral calorie intake. Moreover, HNSCCs cause altered energy metabolism. Malignancies increase the output of reactive oxygen species, tumor glycolysis and the production of lactic acids even in an aerobic milieu (the Warburg effect) [12]. Hence, these conditions should be taken into consideration in the perioperative care of HNSCC patients.

Another condition linked to adverse outcomes and reduced QOL is dysphagia [13,14]. Comprehensive postoperative logopedic evaluation is essential to assess functional outcomes and, when needed, support patients in adjusting to their altered anatomical and physiological conditions. In recent years, preoperative logopedic exercises have also gained recognition as a potentially valuable approach to optimizing postoperative adaptation and recovery [15]. Engaging in swallowing exercises prior to surgery can improve health and function above baseline [16]. In addition, preoperative exercise may also promote neuroplasticity. This enhances patients’ adaptive capacity and shortens recovery time [16]. Similarly, psychological preparation helps patients adapt to the cessation of alcohol and smoking and to cope with their cancer. Preparation can also increase patients’ knowledge and adherence to therapy and might improve overall QOL. These measures can be grouped under the term “pre-rehabilitation” or, for short, “prehabilitation”. Furthermore, they also typically include physiotherapy workout exercises [17,18]. Collectively, these interventions can enhance physical and mental resilience ahead of surgery and evidence supports their role in optimizing outcomes for patients undergoing HNSCC treatment [18,19].

In a multivariate analysis, this study aimed to investigate the impact of malnutrition defined by the Graz Malnutrition Screening (GMS) tool and logopedic, psychological and nutritional MP on QOL, length of stay (LOS) and complication rate (CR) following HNSCC resection and microvascular reconstructive surgery. We introduce the GMS as a new parameter concerning prehabilitation outcomes.

## 2. Methods

### 2.1. Study Design

This cross-sectional study was conducted from August 2024 to October 2025 at the Department of Oral and Maxillofacial Surgery in Salzburg, a high-volume reconstructive expertise center performing more than 100 reconstructive free flap procedures per year. The study protocol was approved on 24 July 2024 by the institutional review board of the ethics committee of Salzburg. This study was conducted in accordance with the principles expressed in the Declaration of Helsinki. Recruitment was conducted during guideline-appropriate post-treatment follow-ups for HNSCC patients.

The patients were given patient-reported outcome questionnaires to evaluate their QOL and psychological prehabilitation. Additionally, retrospective data on the HNSCC, dysphagia, malnutrition, age, sex, relevant diseases, stage of cancer, whether and which types of prehabilitation modalities were performed, treatment, reconstruction, Clavien–Dindo type III and IV, CR and LOS were collected. Swallowing assessment was performed by two speech therapists, and nutritional status was recorded simultaneously by an in-house general practitioner and a clinical dietitian. Malnutrition was defined by a GMS equal or greater than 3.

### 2.2. Inclusion and Exclusion Criteria

Eligible participants were adult German-speaking patients who underwent tumor resection with reconstructive surgery and were able to complete the questionnaire independently. Written consent was obtained from all participants. Patients undergoing radiotherapy and patients in a palliative treatment setting were excluded. In total, 102 patients were included.

### 2.3. Assessment of Quality of Life

Patients completed the German version of the Functional Assessment of Cancer Therapy—Head and Neck (FACT-H&N) questionnaires. License permission to use the FACT-H&N questionnaire was obtained on 10 May 2024. The FACT-H&N consists of the Functional Assessment of Cancer Therapy—General (FACT-G) questionnaire and the head and neck cancer subscale (HNCS). The FACT-G instrument was initially developed by Cella et al. (1993) and currently consists of 27 items, which measure general aspects of QOL relevant to all cancer patients on a 5-point Likert scale (ranging from 0 to 4 points) [20]. The FACT-G can be further divided into 7 items that measure physical well-being, 7 that measure social well-being, 6 that measure emotional well-being and 7 for functional well-being [20]. The 12 items of the HNCS instrument further expand that questionnaire by addressing specific symptoms and concerns of HNSCC patients [21].

If a participant wishes to omit question GS7 on sexual activity, the questionnaire can still be completed in full by checking box Q1. The mean score of the remaining 6 measures is then calculated and multiplied by 7. In total, 37 of the 39 items are then scored. A total of 12 of the FACT-G measures and 4 HNCS items are scored inversely. This means that the question is phrased in a negative way concerning the patient’s QOL, e.g., “I have nausea”, and a response with higher agreement transfers to fewer points in the scoring thus equating to decreased QOL values [20,21].

In total, a score of 0–108 on the FACT-G questionnaire, 0–40 on the HNCS, and 148 on the combined FACT-H&N can be obtained. Additionally, the Trial Outcome Index (TOI) can be scored, comprising the HNCS and the physical and functional well-being domain [20,21].

### 2.4. Data Collection

Retrospectively, data was gathered from our clinical administration system Orbis (Dedalus Healthcare Group, Version 08044207.00000.DACHL, Milano, Italy). Additionally, questions regarding psychological prehabilitation were added to the questionnaire. The treatment concept for MP consisted of nutritional, logopedic and psychological treatments. Patients were considered to have completed MP if they received at least two of the aforementioned treatment protocols.

Mental prehabilitation consisted of education, psychological evaluation, and, in case of mental distress, intensified counseling and mental support from a psychologist. Moreover, patients had been instructed to cease or at least decrease alcohol and smoking prior to surgery, if applicable. Pharmacological supportive therapies had been administered when clinically indicated.

A baseline logopedic evaluation of preexisting voice, speech, swallowing and mouth opening impairment was performed by in-house speech therapists upon HNSCC diagnosis. If necessary, in addition to clinical assessment, a fiberoptic endoscopic evaluation of swallowing was conducted. Based on findings tailored to the relevant preexisting symptoms at hand, patients had been instructed to perform swallowing exercises including the Mendelsohn, chin-tuck and effortful swallowing exercises at least three times a day with 10 repetitions each before surgery.

To prevent aspiration the patient had been advised to adjust food consistency according to the International Dysphagia Diet Standardisation Initiative scale, suitable for the detected dysphagia, to prevent aspiration.

For nutritional support therapy, patients’ height and weight were measured, and body mass index (BMI) was subsequently calculated. Information regarding age, recent weight loss, dysphagia, and the severity of underlying diseases was collected and used to determine the Graz Malnutrition Screening (GMS) score.

As part of nutritional prehabilitation, blood tests were performed to assess serum biomarkers of malnutrition. The analyzed parameters included total serum protein, albumin, prealbumin and transferrin as the main parameters for malnutrition. Other parameters included triglycerides, liver enzymes, including cholinesterase, urea, lactate, total cholesterol, electrolytes, serum creatinine and total lymphocyte count. Based on these assessments, the GMS, clinical parameters and laboratory markers, patients had received nutritional counseling before surgery was performed. Additionally, patients had been advised to consume protein- and calorie-enriched nutritional supplements preoperatively. The products used were all in COMPLETE (all in nutrition GmbH, Vienna, Austria), Fortimel Compact Protein (Danone, Paris, France) and Fresubin Protein Energy (Fresenius Kabi, Bad Homburg, Hesse, Germany).

### 2.5. Statistical Analysis

Data was collected and transferred to Microsoft Excel (Microsoft, Version 2409, Redmond, WA, USA). All statistical analyses were performed using R (R Foundation for Statistical Computing, Version 4.4.3).

Descriptive statistics were calculated for all study variables. Continuous variables are summarized using means and standard deviations, as well as medians and interquartile ranges. Categorical variables are reported as absolute and relative frequencies.

To evaluate the effects of malnutrition and MP on postoperative outcomes, multiple linear regression models were applied. Outcomes included LOS, QOL scores (TOI, FACT-G, FACT-H&N) and postoperative CR. For each outcome, an intercept-only model was first fitted, followed by a model including the primary exposure variable, and finally a multivariable model adjusted for potential confounders.

The multivariable models included the following adjustment variables: age, sex, Union for International Cancer Control (UICC) cancer stage, prior (chemo) radiotherapy, American Society of Anesthesiologists (ASA) score, and relevant comorbidities such as diabetes mellitus, tobacco and alcohol abuse, and BMI as well as preoperative weight loss. BMI and weight loss were only included as confounders for MP. For malnutrition, they were omitted as they are already included in the GMS score.

Cases with missing data in the respective variables were excluded from the analyses (complete-case approach). Statistical significance was defined as *p* < 0.05, with *p* < 0.001 considered highly significant.

## 3. Results

### 3.1. Participants

Of the 102 patients included, 68 were male, while the other 34 were female. The mean age was 59.82 ± 12.27 years (range: 24 to 87) (Table 1).

At the time of surgery, the nutritional parameters, BMI, bodyweight loss and GMS were distributed at 23.94 (±4.32), −1.65 ± 4.87 kg and 3.11 ± 1.45 kg, respectively. In total, 27 patients (26.47%) had a secondary primary cancer or a recurrence of cancer. The average pT stage was 2.52 (±1.15), pN stage was 0.44 (±0.86) and UICC stage was 2.76. (Chemo)radiotherapy following surgery was recorded in 81 (79.40%) members of the study population. Prior to surgery, 45 (44.11%) were smokers, while 32 (31.37%) had stopped smoking. Alcoholism was recorded in 49 (48.04%) patients and former alcoholism in 17 (16.67%). Type II diabetes was present in 7 (6.86%) patients. The average ASA score was 2.51 (±0.558) (Table 1).

### 3.2. FACT H&N Patient-Reported Outcomes (© 1987, 1997 by David Cella, Ph.D. [20,21])

The lowest QOL score, 2.28 (±1.621), was reported for item H&N11. This corresponds to the statement “I can eat solid foods” [21]. Other low-scoring patient-reported outcomes were “My mouth is dry” (H&N 2), “I am able to eat the foods that I like” (H&N 1), “I am able to eat as much food as I want” (H&N 5) and “My voice has its usual quality and strength” (H&N 4) [21].

On the other hand, the outcomes “I have nausea” (GP 2), “I am forced to spend time in bed” (GP 7) and “I am losing hope in the fight against my illness” (GE 3) scored the highest [20] (Table 2). Question GS7 was often omitted. The value was reconstructed according to the official scoring instructions to calculate the social/family well-being subscale total, FACT-G and FACT-H&N scores [20,21].

### 3.3. Malnutrition

**Figure 1:**
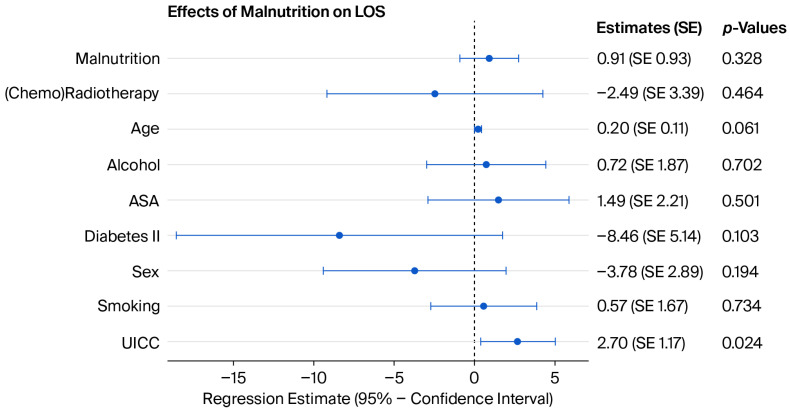
Coefficient plot of the multivariate analysis of the effect of malnutrition on LOS (R^2^ = 0.18). See the Appendix A for more detailed model coefficients.

All patients were screened for malnutrition at the time of HNSCC diagnosis. A state of malnutrition or being at risk thereof was defined as a GMS equal to or greater than 3, which was present in 64 patients (62.75%). We observed a significantly increased LOS in malnourished patients (*p* = 0.0211). However, this finding was not significant in the multivariate analysis (Figure 1).

**Figure 2:**
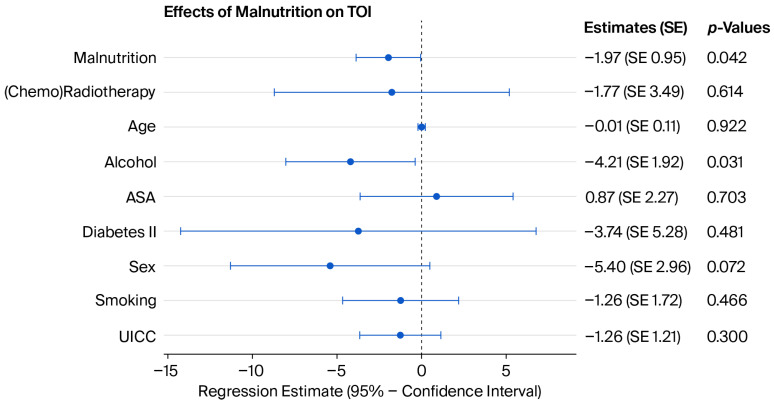
Coefficient plot of the multivariate analysis of the effect of malnutrition on LOS (R^2^ = 0.16). See the Appendix A for more detailed model coefficients.

While a decreased TOI score in malnourished patients could be observed in the univariate model (*p* = 0.0033), neither the FACT-G (*p* = 0.5402) score, FACT-H&N (*p* = 0.0760) score nor the CR were significantly influenced. The association between decreased TOI value and malnutrition remained robust after adjusting for possible confounders (Figure 2).

### 3.4. Multimodal Prehabilitation

**Figure 3:**
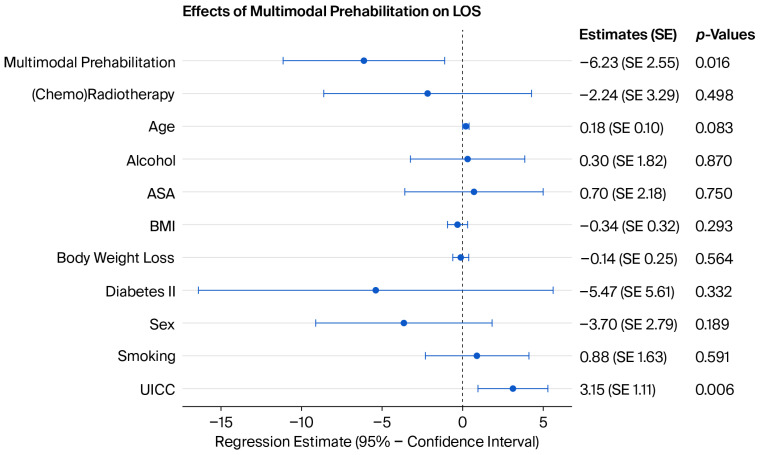
Coefficient plot of the multivariate analysis of the effect of MP on the LOS (R^2^ = 0.251). See the Appendix A for more detailed model coefficients.

A total of 65 patients underwent MP, while 37 did not participate in at least two prehabilitation modes. In the univariate model, MP significantly decreased LOS by 7.4 days. This observation stayed robust even after adjustment for possible confounders (*p* = 0.016) (Figure 3).

**Figure 4:**
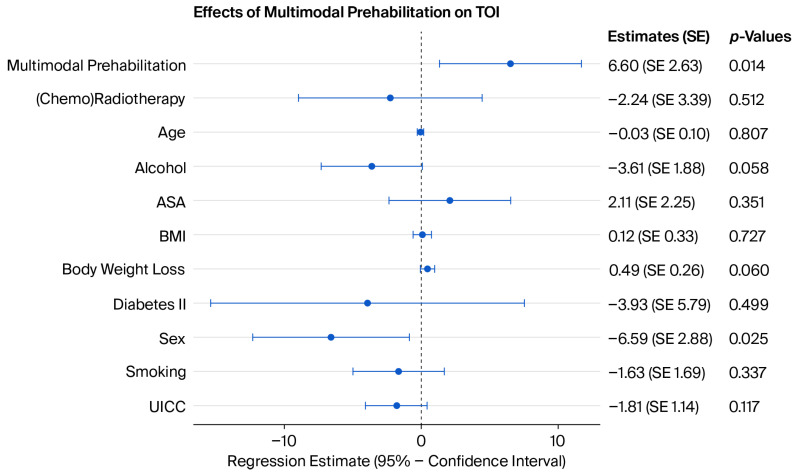
Coefficient plot of the multivariate analysis of MP and TOI (R^2^ = 0.222). See the Appendix A for more detailed model coefficients.

**Figure 5:**
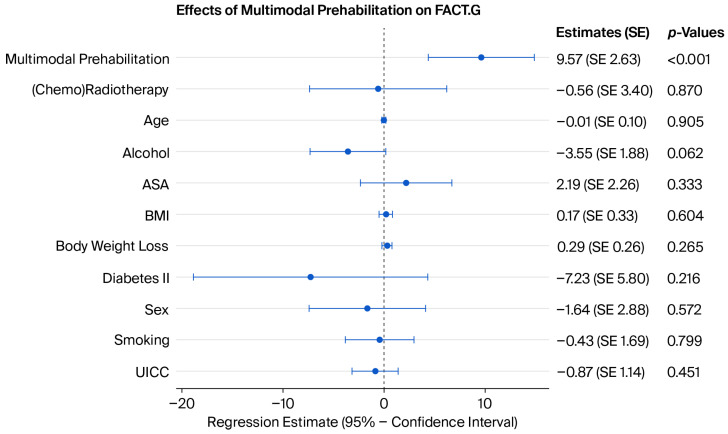
Coefficient plot of the multivariate analysis of MP and the FACT-G score (R^2^ = 0.214). See the Appendix A for more detailed model coefficients.

**Figure 6:**
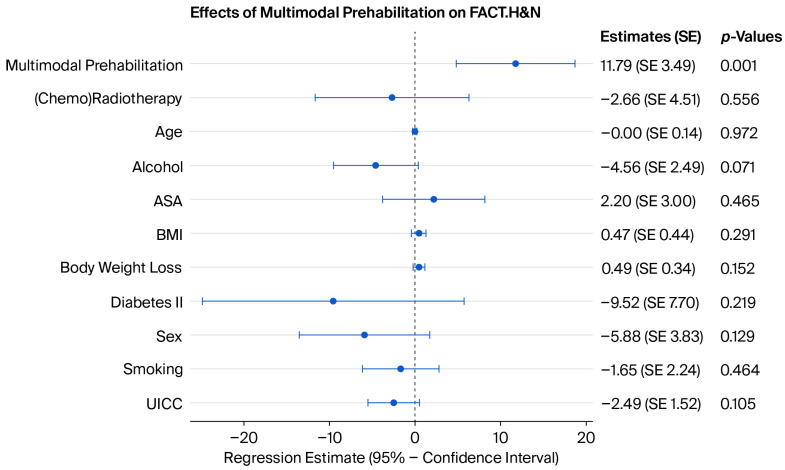
Coefficient plot of the multivariate analysis of MP and the FACT-H&N score (R^2^ = 0.246). See the Appendix A for more detailed model coefficients.

In addition, MP significantly increased the TOI, FACT-G and FACT-H&N in both the univariate and multivariate models (*p* = 0.014, *p* < 0.001 and *p* = 0.001) (Figure 4, Figure 5 and Figure 6). However, there was no association between MP and CR (*p*= 0.948).

## 4. Discussion

Prehabilitation is a well-established supportive treatment concept for cancer patients undergoing surgery. It improves functional capacity [22]. As a result, it may also decrease LOS, CR and post-surgical outcomes by promoting baseline physical, mental and psychological health prior to surgery [23,24]. However, the research on prehabilitation is complicated by the heterogeneity of cancer types and diverse treatment modalities. As palliative or curative treatments, surgery, radiotherapy, and chemotherapy have varying impacts on patients’ functional and psychological outcomes. In addition, there is a variety of prehabilitation treatment protocols, including nutritional supportive measures, logopedic exercises, mental support, detoxification therapy, as well as oral care and physiotherapy workout routines [17,18].

Our data shows promising results for the application of nutritional, psychological and logopedic MP as a possible standard of care for HNSCC surgery preparation protocols. For logopedic prehabilitation, a baseline assessment of existing dysphagia was conducted. Based on these findings, individualized exercises were prescribed according to the patient’s specific condition. In addition, blood tests and clinical evaluations were performed to screen for malnutrition. Then, high-protein and calorie-enriched nutritional supplements were prescribed accordingly. Moreover, mental support, educational and detoxification therapy were offered to the patients.

Patients were considered to have successfully completed MP if they had received at least two of the treatment modalities described above before surgery was performed. This multimodal routine seemed to improve the outcome in terms of LOS, TOI, FACT-G and FACT-H&N scores. Neither high-intensity training nor other structured physiotherapy exercise programs were prescribed, although it is a mode typically featured in prehabilitation. Such models were part of a regimen first described in 1940 by the British army, which invented prehabilitation to prepare soldiers for battle [25]. It has since been historically included in medical pretreatment optimization routines.

However, the primary focus of this study is the role of comprehensive preoperative optimization in the domains of nutrition, logopedics, and psychological prehabilitation, which we hypothesized as the most crucial determinants of postoperative outcomes, and in particular, QOL.

Our data reinforces this hypothesis, as the lowest patient-reported outcome scores were consistently observed in the domains of swallowing, eating, and speech. According to the current literature, these outcomes are frequently reported as poor by HNSCC patients. So et al. (2012) emphasize that QOL generally recovers after the first year post-treatment. Nevertheless, persistent impairments and consequently reduced scores remain in key domains, including swallowing, xerostomia, fatigue, and sticky saliva [26]. Our data is consistent with these observations, except for fatigue, which was generally low-scoring but not among the lowest reported outcomes in our research.

So et al. (2012) noted that fatigue was especially prevalent among patients living with cancer. In total, a 3.6-fold increase can be observed compared to patients alive without cancer [26]. In our study, at the time of the scheduled follow-up visits, the vast majority of patients were free of HNSCC disease. If HNSCC disease was detected, the patients were quickly enrolled in a new curative or palliative treatment and thus excluded from our research on cancer-free HNSCC survivors. The absence of this subgroup accounts for the low incidence of fatigue observed.

Our data also highlight the importance of malnutrition as a possible risk factor in particular. Malnutrition was associated with increased LOS and reduced TOI scores in univariate analyses. However, only the association between malnutrition and TOI remained statistically significant after multivariable adjustment. This attenuation may reflect limited statistical power given the relatively small sample size in relation to the number of covariates included, as well as potential overadjustment. Consequently, the independent contribution of malnutrition to postoperative outcomes remains uncertain and warrants further investigation in larger, prospectively designed studies.

The modest R^2^ values indicate that a substantial proportion of variability in postoperative outcomes remains unexplained, suggesting that additional clinical and biological determinants beyond nutritional status contribute to recovery trajectories in this population.

Notably, the prevalence of malnourished patients in the researched cohort was 62.75%, which is, although on the higher side, generally comparable to the available data reporting between 20 and 80% in HNSCC patients [27,28,29]. The GMS is a validated, highly sensitive (94%) and moderately specific (77%) instrument for the detection of malnutrition in hospitalized patients [30,31]. Likewise, our findings underscore the GMS as a sensitive and possibly valuable tool for malnutrition screening in HNSCC patients and future prehabilitation research, because we believe omitting nutritional prehabilitation in malnourished cachectic patients may confer greater harm than prescribing it in the absence of such conditions.

This is reiterated by our findings on adverse surgical outcomes, which are broadly consistent with the existing literature identifying malnutrition as a major risk factor in cancer surgery. In a prospective multicenter cohort study on 5709 colorectal and gastric cancer patients, Riad and colleagues observed that malnutrition mediated 40% of short-term postoperative mortality and 7% of wound infections [7]. In a retrospective observational study of 92 curatively treated HNSCC patients, Caburet et al. (2020) reported an increase in Clavien–Dindo grade II–V CR among malnourished individuals from 17% to 62% [32]. Likewise, a meta-analysis by Yang and colleagues observed a 1.72-times increase in CR in HNSCC patients presenting with sarcopenia and malnutrition pre-surgery [33]. A plethora of literature confirms the role of malnutrition as a risk factor for adverse effects post-surgery in major HNSCC cases, such as compromised wound healing, increased LOS, higher infection rates and refeeding syndrome [29,34,35,36]. In contrast to other major determinants of clinical outcomes, such as advanced age and multiple comorbidities, this represents a somewhat modifiable risk factor.

The timespan to perform prehabilitation in HNSCC treatment is limited since a timely resection is essential. However, even a short course of nutritional prehabilitation may be sufficient to significantly impact the postoperative outcome, typically seven days to a few weeks of high-calorie, protein-enriched supplementation [37,38]. The Enhanced Recovery After Surgery society guidelines rate the evidence for nutritional prehabilitation as high and strongly recommend it for HNSCC patients undergoing oncological resection and free flap reconstruction [34]. Given evidence supporting the benefit of neoadjuvant immunotherapy in curative treatment of HNSCC, additional time may be available for future presurgical optimization [39].

Adding more complementary modes also increases the effect of prehabilitation. In a systematic research encompassing 31 RCTs, non-RCTs, and observational studies, Seth and colleagues observed that a combination of nutrition and psychoeducation reduced mortality, while nutrition alone only reduced LOS [19]. Immunonutrition is a particular type of nutritional support containing added combinations of the amino acids’ arginine and/or glutamine, omega-3 fatty acids and nucleic acids and it may be particularly beneficial in the perioperative setting. Emerging evidence indicates that perioperative immunonutrition can improve postoperative outcomes further by reducing the risk of postoperative fistula formation [40].

Despite observing a reduced LOS, we did not observe a beneficial effect of MP on the incidence of Clavien–Dindo type III and IV CR. However, we did observe significantly increased post-treatment QOL values in the MP group. This is consistent with findings in the literature concerning nutrition and mental support [19].

Conversely, Semple et al. (2013) did not observe improved QOL or secondary outcomes in anxiety, psychological distress or depression in a meta-analysis concerning psychological interventions [41]. However, they also noted that the data quality was insufficient to refute a benefit [41]. Psychological prehabilitation should include mental support, education, and detoxification, at least addiction counseling, since alcohol and tobacco addictions substantially contribute to worse postoperative outcomes [42,43,44]. Smoking in particular is a well-established risk factor for postoperative complications. It significantly increases the incidence of surgical site infections, wound dehiscence, impaired wound healing and hematoma formation [45,46,47,48]. Simultaneously, it also increases reoperation rates and length of stay in general [44,49]. In this context, psychological prehabilitation can meaningfully improve surgical outcomes by promoting smoking cessation or a substantial reduction in cigarette consumption. Studies indicate that even short periods of preoperative abstinence can yield measurable benefits in reducing certain complications, but a longer period may be more beneficial [44,50].

Whereas psychological prehabilitation emphasizes tobacco and alcohol cessation as well as mental support, logopedic prehabilitation focuses on the evaluation, treatment, and prevention of dysphagia. In a systematic review, no improvement of oral swallowing, QOL, or time to return to function was observed concerning logopedic exercises [51]. On the other hand, according to Brady and colleagues’ systematic review of eight studies featuring 295 HNC patients speech and language therapeutic preoperative interventions have at least a short-term positive effect [52].

Partially in line with our findings, in a meta-analysis of five randomized controlled trials with 423 participants, Vester and colleagues observed only a short-term benefit of logopedic prehabilitation [53]. We observed both types of effects. In addition to potentially aiding immediate postoperative recovery and reducing LOS, our data support long-lasting effects of MP on QOL beyond 6 months after surgery and adjuvant therapies. Concerning short-term benefits, we believe that learning swallowing exercises before surgery in particular helps patients adjust to postoperative logopedic rehabilitation. The long-term benefit we observed in our research might be completely or partially induced by the addition of nutritional and psychological prehabilitation. Notably, there is evidence for such synergistic effects of nutrition and swallowing exercises in the literature [19,38].

In summary, the evidence for nutritional [19,34,37,38], psychological [19,54,55] and logopedic prehabilitation [19,38,52,53,56] is generally very favorable. However, the effect on specific outcomes is disputed in the literature. Moreover, the observed associations may reflect potential synergistic interactions; but, this cannot be formally tested in the present design.

### Strengths and Limitations

The cross-sectional design and marked heterogeneity of the study population constitute principal limitations, preventing causal inference and potentially confounding the observed associations. We sought to mitigate heterogeneity in the study population by performing a multiple regression analysis, adjusting for well-established confounders like age, sex, cancer stage, prior (chemo)radiotherapy, relevant comorbidities such as diabetes, tobacco and alcohol use, and the ASA score. Smoking was evaluated as a potential confounder. Nevertheless, it did not emerge as a statistically significant factor in the multivariable analysis. However, the moderate sample size of 102 patients in relation to the many covariates is a possible limitation and may raise concerns about the model stability. The number of predictors was chosen a priori based on clinical relevance and potential confounding. The ratio of observations to predictors in our model is approximately 10:1, which is within commonly accepted recommendations for multivariable regression analyses. To further assess model stability, we evaluated multicollinearity and found no evidence of concern. Finally, sensitivity analyses using reduced models yielded consistent results regarding the main effect of interest, supporting the robustness of our findings. Unfortunately, the limited sample size also prohibited the subgroup analysis of the multimodal prehabilitation components. In summary, it remains unclear whether the observed effect was induced by psychological prehabilitation, nutritional prehabilitation, logopedic prehabilitation, or a synergistic combination of all three interventions.

Furthermore, the integration of a vast amount of clinical data as well as the use of validated tools is a strength of this study. In that regard, we follow the consensus of experts defining the most important research priorities in the field of prehabilitation. Analyzing the effect of prehabilitation on surgical outcomes while identifying at-risk patients, especially those who would benefit the most from prehabilitation, are highlighted as the two most important research topics [57]. To identify at-risk patients, it is crucial to report known risk factors. We not only reported numerous commonly recognized risk factors but also included them in our multivariate model. According to Raichurkar et al. (2023), there is a need for new screening tools for that population [57]. Evidence shows that one of the most important risk factors for adverse outcomes following cancer surgery is malnutrition [7]. Therefore, we introduced the GMS as a novel screening tool to the field of prehabilitation and HNSCC research.

## 5. Conclusions

Nutrition, logopedic and psychological MP may be a valid presurgical preparation protocol for oncological curative resection and free flap reconstruction in HNSCC patients. However, more research is needed to investigate potential synergistic interactions.

MP addresses crucial conditions that contribute to unfavorable treatment outcomes, such as increased LOS and reduced QOL. In this regard, our findings underscore the negative impact of malnutrition. The GMS may be a promising new tool for screening and identifying at-risk HNSCC patients and should be applied in future clinical prehabilitation and research.

## Figures and Tables

**Table 1:** Baseline characteristics of study population (*n* = 102).

Risk Factor	Mean/Count (±SD)	% of Total
Age	59.82 (±12.27)	-
Sex ratio (m:f)	68:34	-
Body mass index	23.94 (±4.32)	-
Bodyweight loss	−1.65 (±4.87)	-
Graz Malnutrition Screening	3.11 (±1.45)	-
Recurrence/second primary cancer	27	26.47%
pT stage	2.52 (±1.15)	-
pN stage	0.44 (±0.86)	-
UICC stage	2.76 (±1.15)	-
(Chemo)radiotherapy	81	79.40%
Current Smoker	45	44.11%
Former smoker	32	31.37%
Alcoholism	49	48.04%
Former alcoholism	17	16.67%
Diabetes Type II	7	6.86%
ASA score	2.51 (±0.558)	-

**Table 2:** FACT H&N patient-reported QOL scores (*n* = 102) *.

Outcome	Mean (95% CI)	Standard Deviation
GP1 I have a lack of energy ¶	2.80 (2.58–3.02)	0.11
GP2 I have nausea ¶	3.92 (3.85–3.99)	0.03
GP3 Because of my physical condition, I have trouble meeting the needs of my family ¶	3.53 (3.37–3.69)	0.08
GP4 I have pain ¶	3.24 (3.05–3.43)	0.09
GP5 I am bothered by side effects of treatment ¶	3.21 (3.02–3.40)	0.10
GP6 I feel ill ¶	3.60 (3.45–3.75)	0.08
GP7 I am forced to spend time in bed ¶	3.77 (3.64–3.90)	0.07
Physical well-being subscale total (max. 28 pts.)	24.10 (23.40–24.80)	0.36
GS1 I feel close to my friends	3.08 (2.87–3.29)	0.10
GS2 I get emotional support from my family	3.26 (3.04–3.48)	0.11
GS3 I get support from my friends	2.92 (2.66–3.17)	0.13
GS4 My family has accepted my illness	3.56 (3.38–3.74)	0.09
GS5 I am satisfied with family communication about my illness	3.57 (3.40–3.74)	0.09
GS6 I feel close to my partner/main support	3.55 (3.34–3.76)	0.11
GS7 I am satisfied with my sex life §	2.98 (2.76–3.19)	0.11
Social/family well-being subscale total (max. 28 pts.)	22.90 (21.80–24.00)	0.55
GE1 I feel sad ¶	3.30 (3.13–3.47)	0.09
GE2 I am satisfied with how I am coping with my illness	3.17 (2.96–3.38)	0.11
GE3 I am losing hope in the fight against my illness ¶	3.69 (3.56–3.82)	0.07
GE4 I feel nervous ¶	3.29 (3.10–3.48)	0.10
GE5 I worry about dying ¶	3.58 (3.44–3.72)	0.07
GE6 I worry that my condition will get worse ¶	2.99 (2.79–3.19)	0.10
EWB Emotional well-being subscale total (max. 24 pts.)	20.20 (19.50–20.80)	0.34
GF1 I am able to work (including work at home)	2.63 (2.41–2.85)	0.11
GF2 My work (including work at home) is fulfilling	2.82 (2.61–3.03)	0.11
GF3 I am able to enjoy life	2.99 (2.80–3.18)	0.10
GF4 I have accepted my illness	3.11 (2.92–3.30)	0.10
GF5 I am sleeping well	3.02 (2.84.3.20)	0.09
GF6 I am enjoying the things I usually do for fun	3.03 (2.82–3.24)	0.11
GF7 I am content with the quality of my life right now	3.05 (2.87–3.23)	0.09
Functional well-being subscale total (max. 28 pts.)	20.60 (19.70–21.60)	0.48
H&N1 I am able to eat the foods that I like	2.45 (2.17–2.73)	0.14
H&N2 My mouth is dry ¶	2.42 (2.14–2.70)	0.14
H&N3 I have trouble breathing ¶	3.50 (3.29–3.71)	0.10
H&N4 My voice has its usual quality and strength	2.51 (2.25–2.77)	0.13
H&N5 I am able to eat as much food as I want	2.46 (2.19–2.73)	0.13
H&N6 I am unhappy with how my face and neck look ¶	3.02 (2.78–3.26)	0.12
H&N7 I can swallow naturally and easily	2.67 (2.46–2.88)	0.11
H&N10 I am able to communicate with others	3.35 (3.16–3.54)	0.09
H&N11 I can eat solid foods	2.28 (1.96–2.60)	0.16
H&N12 I have pain in my mouth, throat, or neck ¶	2.94 (2.73–3.15)	0.11
Head & neck cancer subscale total (max. 40 pts.)	27.60 (26.20–29.00)	0.69
TOI (max. 96 pts.)	72.13 (69.63–74.63)	1.26
FACT-G (max. 108 pts.)	87.42 (84.97–89.87)	1.23
FACT H&N (max. 148 pts.)	115.18 (111.83–118.53)	1.69

* In all outcomes, a high score equates to a high QOL. Except for the inversely scored questions (marked with ¶), a high score also means agreement with the statement. The questionnaire can be fully completed even when skipping question GS7 (marked with §). The minimum possible score for all outcomes is 0. The maximum score for each outcome is 4, except for the summed-up scores. © 1987, 1997 by David Cella, Ph.D. [20,21].

## Data Availability

The data underlying this article will be shared upon reasonable request to the corresponding author.

## Glossary

ASA	American Society of Anesthesiologists
BMI	Body mass index
CR	Complication rate
FACT-G	Functional Assessment of Cancer Therapy—General
FACT-H&N	Functional Assessment of Cancer Therapy—Head and Neck
GMS	Graz Malnutrition Screening
HNSCC	Head and neck squamous cell carcinoma
LOS	Length of stay
MP	Multimodal prehabilitation
QOL	Quality of life
TOI	Trial outcome index
UICC	Union for International Cancer Control

## Appendix A

**Table A1:** Effects of malnutrition. Model coefficients of multivariate analysis of the effect of malnutrition on LOS (R^2^ = 0.18).

			95% Confidence Interval			
Predictor	Estimate	SE	Lower	Upper	t	*p*
Intercept	3.98	8.34	−12.59	20.55	0.48	0.634
GMS	0.91	0.93	−0.93	2.76	0.98	0.328
Sex	−3.78	2.89	−9.51	1.95	−1.31	0.194
Age	0.20	0.11	−0.01	0.41	1.90	0.061
UICC	2.70	1.17	0.37	5.03	2.30	0.024
ASA	1.49	2.21	−2.89	5.88	0.68	0.501
Smoking	0.57	1.67	−2.76	3.90	0.34	0.734
Alcohol	0.72	1.87	−3.00	4.43	0.38	0.702
Diabetes II	−8.46	5.14	−18.67	1.74	−1.65	0.103
(Chemo)radiotherapy	−2.49	3.39	−9.23	4.25	−0.73	0.464

**Table A2:** Model coefficients of multivariate analysis of the effect of malnutrition on TOI (R^2^ = 0.16).

			95% Confidence Interval			
Predictor	Estimate	SE	Lower	Upper	t	*p*
Intercept	88.32	8.57	71.30	105.33	10.31	<0.001
GMS	−1.97	0.95	−3.87	−0.08	−2.07	0.042
Sex	−5.40	2.96	−11.29	0.49	−1.82	0.072
Age	−0.01	0.11	−0.23	0.21	−0.10	0.922
UICC	−1.26	1.21	−3.65	1.14	−1.04	0.300
ASA	0.87	2.27	−3.64	5.37	0.38	0.703
Smoking	−1.26	1.72	−4.68	2.16	−0.73	0.466
Alcohol	−4.21	1.92	−8.03	−0.39	−2.19	0.031
Diabetes II	−3.74	5.28	−14.22	6.74	−0.71	0.481
(Chemo)radiotherapy	−1.77	3.49	−8.69	5.16	−0.51	0.614

**Table A3:** Effects of MP. Model coefficients of multivariate analysis of the effect of MP on LOS (R^2^ = 0.251).

			95% Confidence Interval			
Predictors	Estimates	Std.-Error	Upper	Lower	t	*p*
Intercept	20.45	11.61	−2.61	43.51	1.76	0.081
Multimodal Prehabilitation	−6.23	2.55	−11.29	−1.17	−2.45	0.016
Age	0.18	0.10	−0.02	0.38	1.75	0.083
Sex	−3.70	2.79	−9.25	1.85	−1.32	0.189
UICC	3.15	1.11	0.95	5.35	2.84	0.006
ASA	0.70	2.18	−3.64	5.04	0.32	0.750
Smoking	0.88	1.63	−2.36	4.12	0.54	0.591
Alcohol	0.30	1.82	−3.31	3.91	0.16	0.870
Diabetes II	−5.47	5.61	−16.61	5.68	−0.97	0.332
(Chemo)radiotherapy	−2.24	3.29	−8.77	4.30	−0.68	0.498
BMI	−0.34	0.32	−0.98	0.30	−1.06	0.293
Bodyweight Loss	−0.14	0.25	−0.64	0.35	−0.58	0.564

**Table A4:** Model coefficients of multivariate analysis of the effect of MP on TOI (R^2^ = 0.222).

			95% Confidence Interval			
Predictors	Estimates	Std.-Error	Upper	Lower	t	*p*
Intercept	76.00	11.98	52.19	99.81	6.34	<0.001
Multimodal Prehabilitation	6.60	2.63	1.37	11.82	2.51	0.014
Age	−0.03	0.10	−0.23	0.18	−0.24	0.807
Sex	−6.59	2.88	−12.32	−0.87	−2.29	0.025
UICC	−1.81	1.14	−4.08	0.46	−1.58	0.117
ASA	2.11	2.25	−2.37	6.59	0.94	0.351
Smoking	−1.63	1.69	−4.98	1.72	−0.97	0.337
Alcohol	−3.61	1.88	−7.34	0.12	−1.92	0.058
Diabetes II	−3.93	5.79	−15.44	7.57	−0.68	0.499
Radiotherapy	−2.24	3.39	−8.98	4.51	−0.66	0.512
BMI	0.12	0.33	−0.54	0.77	0.35	0.727
Bodyweight Loss	0.49	0.26	−0.02	1.00	1.90	0.060

**Table A5:** Model coefficients of multivariate analysis of the effect of MP on FACT-G (R^2^ = 0.214).

			95% Confidence Interval			
Predictors	Estimates	Std.-Error	Upper	Lower	t	*p*
Intercept	79.90	11.99	56.08	103.72	6.66	<0.001
Multimodal Prehabilitation	9.57	2.63	4.34	14.79	3.64	<0.001
Age	−0.01	0.10	−0.22	0.20	−0.12	0.905
Sex	−1.64	2.88	−7.37	4.09	−0.57	0.572
UICC	−0.87	1.14	−3.14	1.41	−0.76	0.451
ASA	2.19	2.26	−2.29	6.68	0.97	0.333
Smoking	−0.43	1.69	−3.78	2.92	−0.26	0.799
Alcohol	−3.55	1.88	−7.28	0.18	−1.89	0.062
Diabetes II	−7.23	5.80	−18.74	4.29	−1.25	0.216
(Chemo)radiotherapy	−0.56	3.40	−7.30	6.19	−0.16	0.870
BMI	0.17	0.33	−0.49	0.83	0.52	0.604
Bodyweight Loss	0.29	0.26	−0.22	0.80	1.12	0.265

**Table A6:** Model coefficients of multivariate analysis of the effect of MP on FACT H&N (R^2^ = 0.246).

			95% Confidence Interval			
Predictors	Estimates	Std.-Error	Upper	Lower	t	*p*
Intercept	108.93	15.93	77.29	140.57	6.84	<0.001
Multimodal Prehabilitation	11.79	3.49	4.84	18.73	3.37	0.001
Age	−0.00	0.14	−0.28	0.27	−0.04	0.972
Sex	−5.88	3.83	−13.49	1.74	−1.53	0.129
UICC	−2.49	1.52	−5.51	0.53	−1.64	0.105
ASA	2.20	3.00	−3.75	8.15	0.73	0.465
Smoking	−1.65	2.24	−6.10	2.80	−0.73	0.464
Alcohol	−4.56	2.49	−9.51	0.40	−1.83	0.071
Diabetes II	−9.52	7.70	−24.81	5.77	−1.24	0.219
(Chemo)radiotherapy	−2.66	4.51	−11.63	6.30	−0.59	0.556
BMI	0.47	0.44	−0.41	1.34	1.06	0.291
Bodyweight Loss	0.49	0.34	−0.19	1.18	1.45	0.152

## References

[1] Tannock I.F., Pe M.L., Booth C.M., Brundage M., Cherny N.I., Coens C., Eisenhauer E.A., Geissler J., Giesinger J.M., Gyawali B. (2025). Importance of responder criteria for reporting health-related quality-of-life data in clinical trials for advanced cancer: Recommendations of Common Sense Oncology and the European Organisation for Research and Treatment of Cancer. Lancet Oncol..

[2] Mücke T., Wolff K.D., Wagenpfeil S., Mitchell D.A., Hölzle F. (2010). Immediate microsurgical reconstruction after tumor ablation predicts survival among patients with head and neck carcinoma. Ann. Surg. Oncol..

[3] de Vicente J.C., Rodríguez-Santamarta T., Rosado P., Peña I., de Villalaín L. (2012). Survival After Free Flap Reconstruction in Patients with Advanced Oral Squamous Cell Carcinoma. J. Oral Maxillofac. Surg..

[4] Galviz Tabares B., Ruiz Geithner C.M., Pierpoline J., Mosquera C. (2025). Long-Term Functional Outcomes of Free Flaps Versus Locoregional Flaps in Soft Tissue Reconstruction for Oral Cavity Cancer: A Systematic Review. J. Craniofac. Surg..

[5] Højvig J.H., Pedersen N.J., Charabi B.W., Wessel I., Jensen L.T., Nyberg J., Mayman-Holler N., Kehlet H., Bonde C.T. (2020). Microvascular reconstruction in head and neck cancer—Basis for the development of an enhanced recovery protocol. JPRAS Open.

[6] Kapoor D., Cleere E.F., Hurley C.M., de Blacam C., Theopold C.F.P., Beausang E. (2023). Frailty as a predictor of adverse outcomes in head and neck reconstruction: A systematic review. J. Plast. Reconstr. Aesthetic Surg..

[7] Riad A., Knight S.R., Ghosh D., Kingsley P.A., Lapitan M.C., Parreno-Sacdalan M.D., Sundar S., Qureshi A.U., Valparaiso A.P., Pius R. (2023). Impact of malnutrition on early outcomes after cancer surgery: An international, multicentre, prospective cohort study. Lancet Glob. Health.

[8] Fearon K., Strasser F., Anker S.D., Bosaeus I., Bruera E., Fainsinger R.L., Jatoi A., Loprinzi C., MacDonald N., Mantovani G. (2011). Definition and classification of cancer cachexia: An international consensus. Lancet Oncol..

[9] Penet M.F., Bhujwalla Z.M. (2015). Cancer cachexia, recent advances, and future directions. Cancer J..

[10] Guerra-Londono C.E., Cata J.P., Nowak K., Gottumukkala V. (2024). Prehabilitation in Adults Undergoing Cancer Surgery: A Comprehensive Review on Rationale, Methodology, and Measures of Effectiveness. Curr. Oncol..

[11] Cruz-Jentoft A.J., Baeyens J.P., Bauer J.M., Boirie Y., Cederholm T., Landi F., Martin F.C., Michel J.-P., Rolland Y., Schneider S.M. (2010). Sarcopenia: European consensus on definition and diagnosis: Report of the European Working Group on Sarcopenia in Older People. Age Ageing.

[12] Liberti M.V., Locasale J.W. (2016). The Warburg Effect: How Does it Benefit Cancer Cells?. Trends Biochem. Sci..

[13] Yifru T.A., Kisa S., Dinegde N.G., Atnafu N.T. (2021). Dysphagia and its impact on the quality of life of head and neck cancer patients: Institution-based cross-sectional study. BMC Res. Notes.

[14] Chen A.Y., Frankowski R., Bishop-Leone J., Hebert T., Leyk S., Lewin J., Goepfert H. (2001). The Development and Validation of a Dysphagia-Specific Quality-of-Life Questionnaire for Patients with Head and Neck Cancer: The M. D. Anderson Dysphagia Inventory. Arch. Otolaryngol. Head. Neck Surg..

[15] Dietz A., Taylor K., Bayer O., Singer S., Langer T. (2025). Evidence-based guideline diagnosis, treatment, prevention and aftercare of oropharyngeal and hypopharyngeal carcinoma [S3-Leitlinie zur Diagnostik, Therapie, Prävention und Nachsorge des Oro- und Hypopharynxkarzinoms]. Ger. Med. Sci..

[16] Loewen I., Jeffery C.C., Rieger J., Constantinescu G. (2021). Prehabilitation in head and neck cancer patients: A literature review. J. Otolaryngol. Head. Neck Surg..

[17] Groen L.C.B., de Vries C.D., Mulder D.C., Daams F.D., Bruns E.R.J., Helmers R., Schreurs H.W.H. (2025). Multimodal Prehabilitation in Head and Neck Cancer Patients Undergoing Surgery: A Feasibility Study. J. Hum. Nutr. Diet..

[18] Demurtas S., Cena H., Benazzo M., Gabanelli P., Porcelli S., Preda L., Bortolotto C., Bertino G., Mauramati S., Veneroni M.V. (2024). Head and Neck Cancer (HNC) Prehabilitation: Advantages and Limitations. J. Clin. Med..

[19] Seth I., Bulloch G., Qin K.R., Xie Y., Sebastian B., Liew H., Rozen W.M., Lee C.H.A. (2024). Pre-rehabilitation interventions for patients with head and neck cancers: A systematic review and meta-analysis. Head Neck.

[20] Cella D.F., Tulsky D.S., Gray G., Sarafian B., Linn E., Bonomi A., Silberman M., Yellen S.B., Winicour P., Brannon J. (1993). The Functional Assessment of Cancer Therapy scale: Development and validation of the general measure. J. Clin. Oncol..

[21] List M.A., D’Antonio L.L., Cella D.F., Siston A., Mumby P., Haraf D., Vokes E. (1996). The Performance Status Scale for Head and Neck Cancer Patients and the Functional Assessment of Cancer Therapy-Head and Neck Scale. A Study of Utility and Validity. Cancer.

[22] Molenaar C.J., van Rooijen S.J., Fokkenrood H.J., Roumen R.M., Janssen L., Slooter G.D. (2022). Prehabilitation versus no prehabilitation to improve functional capacity, reduce postoperative complications and improve quality of life in colorectal cancer surgery. Cochrane Database Syst. Rev..

[23] Zhao B., Zhang T., Chen Y., Zhang C. (2023). Effects of unimodal or multimodal prehabilitation on patients undergoing surgery for esophagogastric cancer: A systematic review and meta-analysis. Support. Care Cancer.

[24] Guo Y., Ding L., Miao X., Jiang X., Xu T., Xu X., Zhu S., Xu Q., Hu J. (2022). Effects of prehabilitation on postoperative outcomes in frail cancer patients undergoing elective surgery: A systematic review and meta-analysis. Support. Care Cancer.

[25] Bargnes V., Davidson S., Talbot L., Jin Z., Poppers J., Bergese S.D. (2024). Start Strong, Finish Strong: A Review of Prehabilitation in Cardiac Surgery. Life.

[26] So W.K., Chan R.J., Chan D.N., Hughes B.G., Chair S.Y., Choi K.C., Chan C.W. (2012). Quality-of-life among head and neck cancer survivors at one year after treatment—A systematic review. Eur. J. Cancer.

[27] Cristofaro M.G., Barca I., Ferragina F., Novembre D., Ferro Y., Pujia R., Montalcini T. (2021). The health risks of dysphagia for patients with head and neck cancer: A multicentre prospective observational study. J. Transl. Med..

[28] Wallmander C., Bosaeus I., Silander E., Berg M., Cange H.H., Nyman J., Hammerlid E. (2025). Malnutrition in patients with advanced head and neck cancer: Exploring the Global Leadership Initiative on Malnutrition (GLIM) criteria, energy balance and health-related quality of life. Clin. Nutr. ESPEN.

[29] Reed W.T., Jiang R., Ohnuma T., Kahmke R.R., Pyati S., Krishnamoorthy V., Raghunathan K., Osazuwa-Peters N. (2024). Malnutrition and Adverse Outcomes After Surgery for Head and Neck Cancer. JAMA Otolaryngol. Head. Neck Surg..

[30] Roller R.E., Eglseer D., Eisenberger A., Wirnsberger G.H. (2016). The Graz Malnutrition Screening (GMS): A new hospital screening tool for malnutrition. Br. J. Nutr..

[31] Sahin N., Tek N.A. (2022). Validity of the Graz Malnutrition Screening as an indicator of malnutrition in hospitalized patients. Nutr. Clin. Pract..

[32] Caburet C., Farigon N., Mulliez A., Mom T., Boirie Y., Gilain L., Saroul N. (2020). Impact of nutritional status at the outset of assessment on postoperative complications in head and neck cancer. Eur. Ann. Otorhinolaryngol. Head Neck Dis..

[33] Yang D., Su L., Zhang L., Zhang Y., Li Y., Huang T., Huang X. (2024). Sarcopenia predicts postoperative complications in head and neck cancer: A systematic review and meta-analysis. Eur. Arch. Otorhinolaryngol..

[34] Dort J.C., Farwell D.G., Findlay M., Huber G.F., Kerr P., Shea-Budgell M.A., Simon C., Uppington J., Zygun D., Ljungqvist O. (2017). Optimal Perioperative Care in Major Head and Neck Cancer Surgery with Free Flap Reconstruction: A Consensus Review and Recommendations from the Enhanced Recovery After Surgery Society. JAMA Otolaryngol. Head Neck Surg..

[35] Alhallak R., Estoup E., Adelou S., Becaud J., Barrat A., Farigon N., Le Bacquer O., Boirie Y., Puechmaille M., Mom T. (2025). Refeeding syndrome in head and neck cancers: A risk in the prehabilitation of patients?. Support. Care Cancer.

[36] Modesti C.L., Mattavelli D., Testa G., Tofani L., Piazza C. (2025). The impact of immunonutrition in head and neck cancer surgery: A systematic review with meta-analysis. Acta Otorhinolaryngol. Ital..

[37] Shen Y., Cong Z., Ge Q., Huang H., Wei W., Wang C., Jiang Z., Wu Y. (2024). Effect of nutrition-based prehabilitation on the postoperative outcomes of patients with esophagogastric cancer undergoing surgery: A systematic review and meta-analysis. Cancer Med..

[38] De Pasquale G., Mancin S., Matteucci S., Cattani D., Pastore M., Franzese C., Scorsetti M., Mazzoleni B. (2023). Nutritional prehabilitation in head and neck cancer: A systematic review of literature. Clin. Nutr. ESPEN.

[39] Uppaluri R., Haddad R.I., Tao Y., Le Tourneau C., Lee N.Y., Westra W., Chernock R., Tahara M., Harrington K.J., Klochikhin A.L. (2025). Neoadjuvant and Adjuvant Pembrolizumab in Locally Advanced Head and Neck Cancer. N. Engl. J. Med..

[40] Howes N., Atkinson C., Thomas S., Lewis S.J. (2018). Immunonutrition for patients undergoing surgery for head and neck cancer. Cochrane Database Syst. Rev..

[41] Semple C., Parahoo K., Norman A., McCaughan E., Humphris G., Mills M. (2013). Psychosocial interventions for patients with head and neck cancer. Cochrane Database Syst. Rev..

[42] Chen S.Y., Massa S., Mazul A.L., Kallogjeri D., Yaeger L., Jackson R.S., Zevallos J., Pipkorn P. (2020). The association of smoking and outcomes in HPV-positive oropharyngeal cancer: A systematic review. Am. J. Otolaryngol..

[43] Dong B., Yu D., Jiang L., Liu M., Li J. (2023). Incidence and risk factors for postoperative delirium after head and neck cancer surgery: An updated meta-analysis. BMC Neurol..

[44] Wong C., Mohamad Asfia S.K.B., Myles P.S., Cunningham J., Greenhalgh E.M., Dean E., Doncovio S., Briggs L., Graves N., McCaffrey N. (2025). Smoking and Complications After Cancer Surgery: A Systematic Review and Meta-Analysis. JAMA Netw. Open.

[45] Crippen M.M., Patel N., Filimonov A., Brady J.S., Merchant A.M., Baredes S., Park R.C.W. (2019). Association of Smoking Tobacco with Complications in Head and Neck Microvascular Reconstructive Surgery. JAMA Facial Plast. Surg..

[46] Garip M., Van Dessel J., Grosjean L., Politis C., Bila M. (2021). The impact of smoking on surgical complications after head and neck reconstructive surgery with a free vascularised tissue flap: A systematic review and meta-analysis. Br. J. Oral Maxillofac. Surg..

[47] Zhao E.H., Nishimori K., Brady J., Siddiqui S.H., Eloy J.A., Baredes S., Park R.C.W. (2018). Analysis of Risk Factors for Unplanned Reoperation Following Free Flap Surgery of the Head and Neck. Laryngoscope.

[48] Sørensen L.T. (2012). Wound Healing and Infection in Surgery: The Clinical Impact of Smoking and Smoking Cessation: A Systematic Review and Meta-analysis. Arch. Surg..

[49] Day T., Tooze J., Sterba K., Hatcher J., Weaver K., Fitzgerald N., Sullivan C., Alberg A., Carpenter M. (2016). Tobacco use and surgical outcomes in patients with head and neck cancer. Head Neck.

[50] Tang E., Rodriguez R.M., Srivastava A., Malhan R., Laksono I., Yan E., Englesakis M., Wong J., Chung F. (2025). Impact of short duration smoking cessation on post-operative complications: A systematic review and meta-analysis. J. Clin. Anesth..

[51] Perry A., Lee S.H., Cotton S., Kennedy C. (2016). Therapeutic exercises for affecting post-treatment swallowing in people treated for advanced-stage head and neck cancers. Cochrane Database Syst. Rev..

[52] Brady R., McSharry L., Lawson S., Regan J. (2022). The impact of dysphagia prehabilitation on swallowing outcomes post-chemoradiation therapy in head and neck cancer: A systematic review. Eur J Cancer Care.

[53] Vester S., Muhr A., Meier J., Süß C., Kummer P., Künzel J. (2023). Prehabilitation of dysphagia in the therapy of head and neck cancer—A systematic review of the literature and evidence evaluation. Front. Oncol..

[54] Ikeda T., Toyama S., Harada T., Noma K., Hamada M., Kitagawa T. (2024). Effectiveness of prehabilitation during neoadjuvant therapy for patients with esophageal or gastroesophageal junction cancer: A systematic review. Esophagus.

[55] Tsimopoulou I., Pasquali S., Howard R., Desai A., Gourevitch D., Tolosa I., Vohra R. (2015). Psychological Prehabilitation Before Cancer Surgery: A Systematic Review. Ann. Surg. Oncol..

[56] Ekici E., Yüzbaşıoğlu Ü., Özkeskin M., Özden F. (2025). Effects of exercise on head and neck cancer surgery: A systematic review of randomized controlled trials. Eur. Arch. Otorhinolaryngol..

[57] Raichurkar P., Denehy L., Solomon M., Koh C., Pillinger N., Hogan S., McBride K., Carey S., Bartyn J., Hirst N. (2023). Research Priorities in Prehabilitation for Patients Undergoing Cancer Surgery: An International Delphi Study. Ann. Surg. Oncol..

